# Effects of dynamic and rigid implantation on biomechanical characteristics of different sagittal alignment lumbar after single- or double-level spinal fixations: a finite-element modeling study

**DOI:** 10.1186/s40001-023-01475-y

**Published:** 2023-12-11

**Authors:** Wei Wang, Chao Kong, Fumin Pan, Xueqing Wu, Baoqing Pei, Shibao Lu

**Affiliations:** 1 Department of Orthopedics, Xuanwu Hospital, Capital Medical University, No.45 Changchun Street, Xicheng District, Beijing, 10053 China; 2 National Clinical Research Center for Geriatric Diseases, Beijing, 10053 China; 3 Beijing Key Laboratory for Design and Evaluation Technology of Advanced Implantable & Interventional Medical Devices, Beijing Advanced Innovation Center for Biomedical Engineering, School of Biological Science and Medical Engineering, Beihang University, Beijing, 100083 China

**Keywords:** Coflex interspinous stabilization, Lumbar fusion, Biomechanical characteristics, Sagittal alignment, Adjacent segment degeneration

## Abstract

**Background:**

Although it is critical to understand the accelerated degeneration of adjacent segments after fusion, the biomechanical properties of the spine have not been thoroughly studied after various fusion techniques. This study investigates whether four Roussouly’s sagittal alignment morphotypes have different biomechanical characteristics after different single- or double-level spinal fixations.

**Methods:**

The parametric finite element (FE) models of Roussouly’s type (1–4) were developed based on the radiological data of 625 Chinese community population. The four Roussouly's type models were reassembled into four fusion models: single-level L4–5 Coflex fixation model, single-level L4–5 Fusion (pedicle screw fixation) model, double-level Coflex (L4–5) + Fusion (L5–S1) model, and double-level Fusion (L4–5) + Fusion (L4–5) model. A pure moment of 7.5 Nm was applied to simulate the physiological activities of flexion, extension, lateral bending and axial rotation.

**Results:**

Both single-level and double-level spinal fixation had the greatest effect on lumbar range of motion, disc pressure, and annulus fibrosis stress in flexion, followed by lateral bending, extension, and axial rotation. In all models, the upper adjacent segment was the most influenced by the implantation and bore the most compensation from the fixed segment. For Type 2 lumbar, the L4–L5 Coflex effectively reduced the disc pressure and annulus fibrosis stress in adjacent segments compared to the L4–L5 Fusion. Similarly, the L4–L5 Coflex offered considerable advantages in preserving the biomechanical properties of adjacent segments for Type 1 lumbar. For Type 4 lumbar, the L4–L5 Coflex did not have superiority over the L4–L5 Fusion, resulting in a greater increase in range of motion at adjacent segments in flexion and extension. The difference between the two fixations was not apparent in Type 3 lumbar. Compared to the single-level Fusion, the changes in motion and mechanics of the lumbar increased after both the double-level Coflex + Fusion and Fusion + Fusion fixations, while the differences between two double-level fixation methods on adjacent segments of the four lumbar models were similar to that of the single-level fixation.

**Conclusion:**

Type 3 and Type 4 lumbar have good compensatory ability and therefore allow for a wider range of surgical options, whereas surgical options for small lordotic Type 1 and Type 2 lumbar are more limited and severe.

**Supplementary Information:**

The online version contains supplementary material available at 10.1186/s40001-023-01475-y.

## Introduction

Although lumbar fusion with posterior pedicle fixation is the gold standard for degenerative lumbar diseases, the incidence rate of secondary accelerated degeneration of adjacent segments is up to 25% [1]. Spine fusions alter the biomechanical environment within the vertebral body, impair blood oxygen and nutrient supply, and result in postoperative problems in adjacent segments [2]. In some circumstances, traditional lumbar fusion has intrinsic limitations, such as a longer operating time, increased blood loss, and enhanced stiffness, which may lead to overtreatment of the patient. The high frequency of secondary accelerated degenerative diseases at adjacent levels after lumbar fusion remains a challenge for orthopedic surgeons.

Coflex interspinous stabilization is a common non-fusion approach that aims to provide adequate stability while delaying the degeneration of adjacent segments by preserving partially segmental motion and allowing for physiological load transmission [3–5]. Several previous studies found the Coflex technique to be safe and effective for treating lumbar disorders [6, 7]. For a minimum of 8 years, Zheng et al. [5] found no difference in the patient’s radiographic results between single-level Coflex stabilization and traditional posterior fusion. The advantage of Coflex stabilization over traditional fusions, on the other hand, remained unclear.

**Fig. 1:**
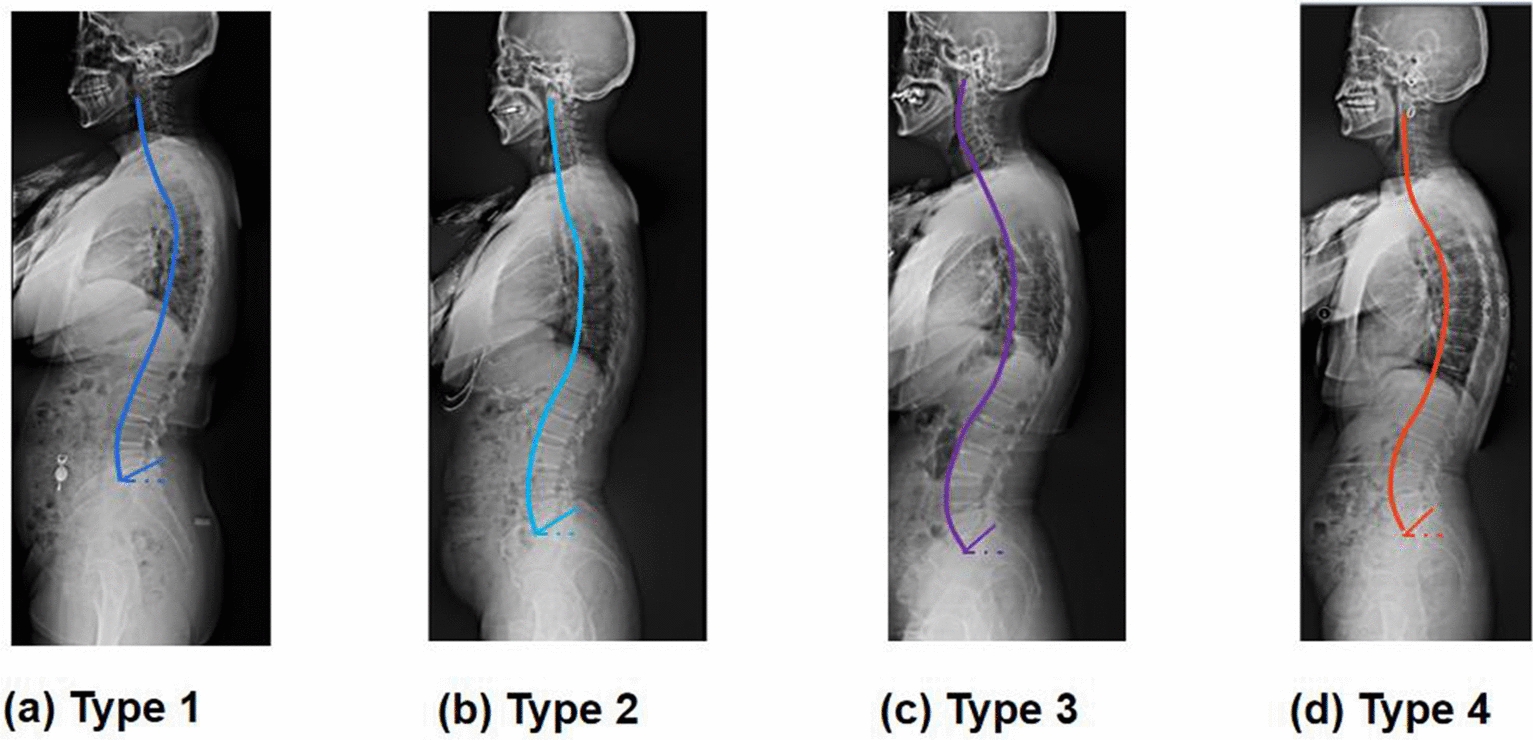
Roussouly classification of the sagittal alignment of the spine based on sacral slope. **a** Type 1 (sacral slope < 35°), a long thoracolumbar kyphosis and a short lumbar lordotic curve; **b** type 2 (sacral slope < 35°), a flat lumbar spine having a fat back appearance; **c** type 3 (35° < sacral slope < 45°), an almost equal length of the kyphotic and lumbar lordosis curves; and **d** type 4 (35° < sacral slope), a long lumbar lordosis and a shorter kyphosis

The spine is made up of functional units and has a multi-segment structure. Over the last 15 years, epidemiological and clinical studies have shown that the sagittal S-shaped curvature of the human spine eliminates energy expenditure of the back muscles while preserving balance and stability [8–11]. The sagittal alignment of the spine is a recently developed concept to understand the mechanical equilibrium process and develop therapeutic strategies for a variety of spinal diseases [11–13]. Four types of sagittal spine alignment were first proposed by Roussouly et al. [9] based on the sacral slope and the spine shape in 2005 (Fig. 1). Notably, each type of spine has a unique mechanical transmission and equilibrium pattern, which is related to pathological evolution and postoperative mechanical complications. Spinal morphology is important in the prevention of spinal dysfunction and the assessment of clinical spinal surgery outcomes. The optimum spinal surgery treatment should relieve focal segmental sickness while rebuilding lumbar stability. Simultaneously, surgical techniques should seek to minimize the changes in biomechanical characteristics of the lumbar, particularly in adjacent segments. The biomechanical properties of the different sagittal shapes of the lumbar have not been thoroughly studied after various fusion techniques, although it is crucial to understand the accelerated degradation of adjacent segments after fusion.

Therefore, this study developed parametric FE models of Roussouly’s type (1–4) to investigate whether different Roussouly sagittal alignment morphotypes have various biomechanical characteristics after different single or double-level spinal fixations, particularly adjacent segments. This study aimed to propose a preliminary assessment approach for studying the effect of implant devices on the biomechanical response of the spine with different sagittal alignment morphotypes. Using the purpose-developed models, we addressed: (1) how do the dynamic Coflex and traditional Fusion fixation alter the biomechanical responses of the lumbar after the single or double-level spinal fixations under the daily loading conditions? (2) what is the difference in the biomechanical responses of four Roussouly sagittal alignment lumbar morphotypes under the daily loading conditions? (3) Which morphotype of the lumbar does the dynamic Coflex have the superiority over the traditional Fusion fixation?

## Method and materials

### Construction of four type sagittal models

**Fig. 2:**
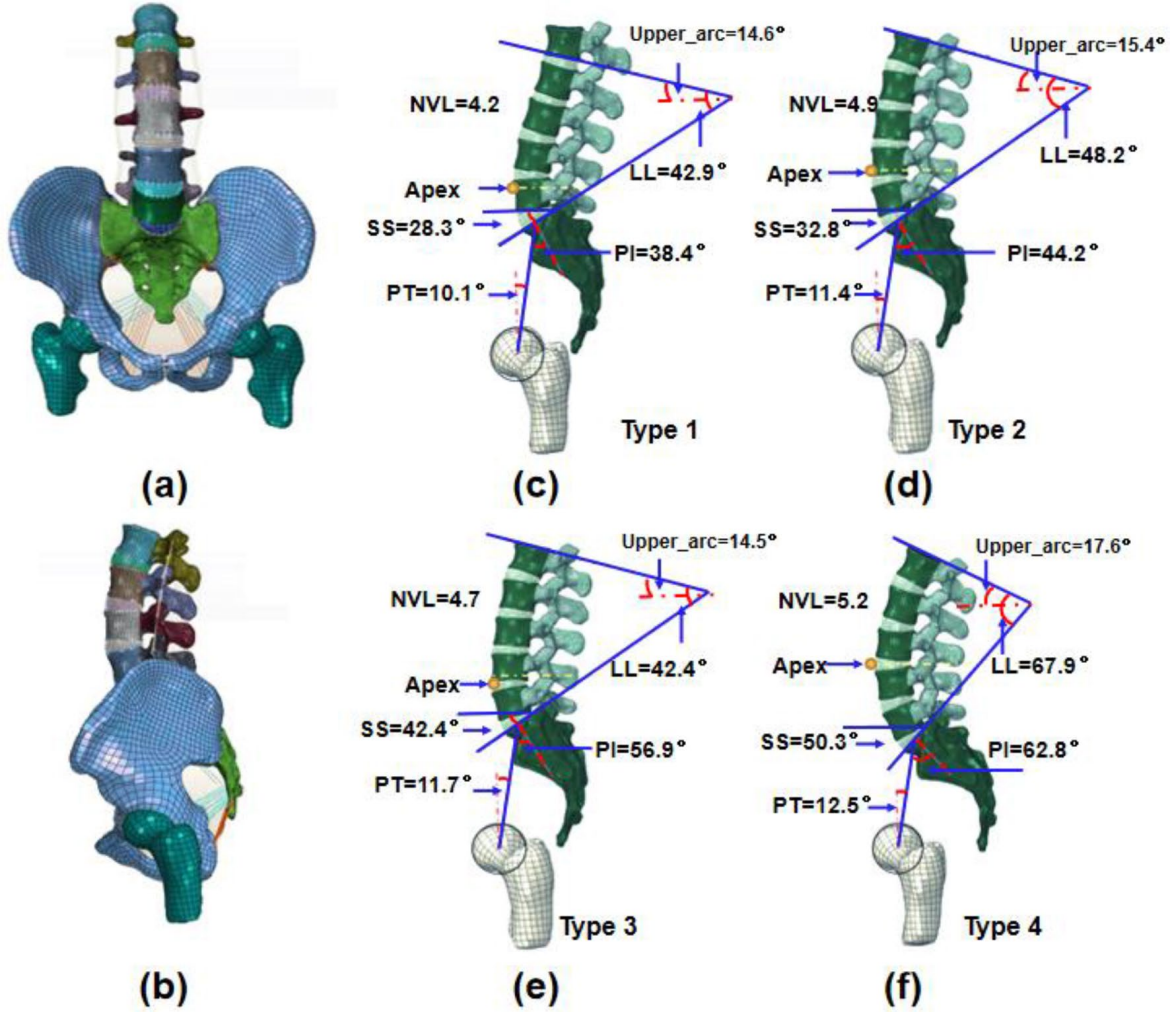
**a** Front and **b** lateral views of the FE model of the lumbo-pelvis; four Roussouly’s type models: **c** type 1 model, **d** type 2 model, **e** type 3 model, and **f** type 4 model. PI: pelvic incidence; PT: pelvic tilt; SS: sacral slope; LL: lumbar lordosis; Apex: the apex of the lordosis; NVL: the number of vertebrae in the lordosis

The parametric FE models of Roussouly's types were recreated using CT images of the human donor, the details of which were disclosed in our previous study [14], as shown in Fig. 2a, b. The lumbar–pelvic parameters of the four Roussouly’s type models were assumed according to sagittal spinopelvic morphotypes of 162 Chinese people in a standardized standing posture (Fig. 2c–f). The lumbar–pelvic parameters included pelvic parameters (pelvic incidence (PI), pelvic title (PT), and sacral slope (SS)), lumbar parameters (lumbar lordosis (LL), Apex, upper arc, and the number of vertebrae in the lordosis (NVL)). The nucleus pulposus (NP), annulus fibrous (AF), and endplates comprised the IVD. The AF was separated into seven layers, including the matrix and fibrous layers. In the crossing-patterned directions, a single fibrous layer was rebuilt using two-family fibers. The absolute values of the fiber angles rose from the ventral to the dorsal Sections (24° to 46°) [15, 16]. The annulus collagen fibers and ligaments, namely anterior (ALL) and posterior longitudinal ligaments (PLL), flava (FL), supraspinous (SSL), interspinous (ISL), and capsular ligaments (FC), were also incorporated into the model. The facet joint surfaces were modeled using frictionless surface-to-surface contact.

### Construction of four type sagittal models after different fusion

**Fig. 3:**
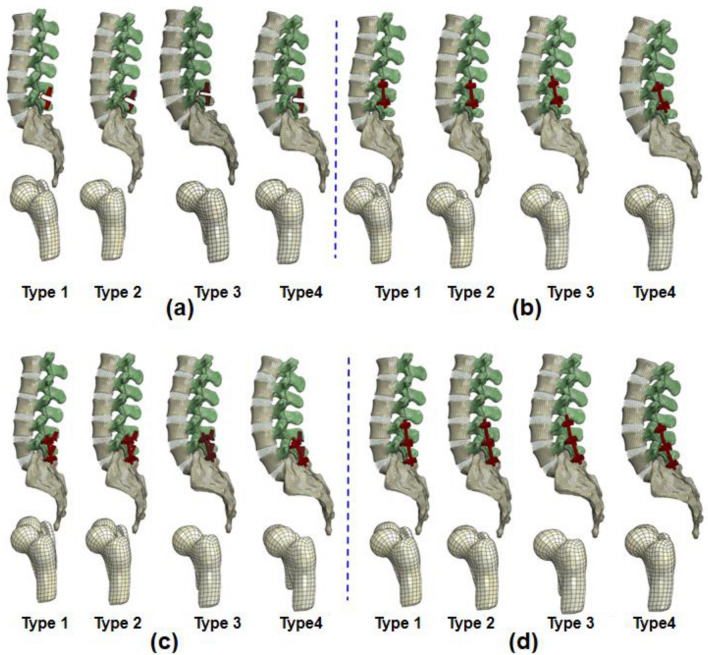
Finite element lumbopelvic models of four Roussouly types after the single- and double-level fixation. **a** single-level L4–5 Coflex fixation model, **b** L4–L5 Fusion: single-level L4–5 pedicle screw fixation model, **c** Coflex + Fusion: double-level L4–5 Coflex + L5–S1 pedicle screw fixation model, and **d** Fusion + Fusion: double-level L4–5Coflex + L5–S1 pedicle screw fixation model

The dynamic Coflex and pedicle screw rod Implantation were modeled and then assembled into four lumbopelvic models (Fig. 3). Four types of lumbopelvic models were reconstructed into single-segment and double-segment fusion models, with a total of 16: L4–L5 Coflex: single-level L4–5 Coflex fixation model (Fig. 3a); L4–L5 Fusion: single-level L4–5 pedicle screw fixation model (Fig. 3b); Coflex + Fusion: double-level L4–5 Coflex + L5–S1 pedicle screw fixation model (Fig. 3c); Fusion + Fusion: double-level L4–5Coflex + L5–S1 pedicle screw fixation model (Fig. 3d).

### Materials and mesh convergence

The material properties of each part of the model are shown in Table 1 [16–19]. Mooney–Rivlin constitutive law was assumed to describe the fluid-like behavior of the NP and annulus matrix. A nonlinear function was used to describe the tensile stress–strain of collagen fibers [19]. The surface of facet joints was considered to be in hard contact with a friction coefficient of 0.15. Elastic isotropy (Young modulus of 35 MPa) was described for facet cartilage layers with an initial clearance of 0.5 mm [20].

**Table 1:** Material properties of the model

Structure	Young’s modulus (MPa)	Poisson’s ratio
Vertebrae		
Cortical bone	E_x_ = 11,300; E_y_ = 11,30; E_z_ = 22,000; G_x_ = 3,800; G_y_ = 5,400; G_z_ = 5,400	ν_xy_ = 0.484; ν_xz_ = 0.203; ν_yz_ = 0.203
Cancellous bone	E_x_ = 140; E_y_ = 140; E_z_ = 200; G_x_ = 48.3; G_y_ = 48.3; G_z_ = 48.3	ν_xy_ = 0.45; ν_xz_ = 0.315; ν_yz_ = 0.315
Posterior elements	3500	0.250
Pelvis–femur		
Cortical bone	15,000	0.30
Cancellous bone	100	0.20
Disc	14.0	13.9
Nucleus pulposus	Hyperelastic, Mooney–Rivlin: C10 = 0.18, C01 = 0.045	
Annulus matrix	Hyperelastic, Mooney–Rivlin: C10 = 0.12, C01 = 0.03	
Fiber	Shirazi-adl's stress–strain curve	
Endplate	3000	0.25
Ligaments		
ALL	7.8(< 12.0%), 20.0(> 12.0%)	0.40
PLL	10.0(< 11.0%), 20.0(> 11.0%)	0.30
SSL	8.0(< 20.0%), 15.0(> 20.0%)	0.30
ISL	10.0(< 14.0%), 11.6(> 14.0%)	0.30
LF	15.8(< 6.2%), 19.5(> 6.2%)	0.30
TL	10.0(< 18.0%), 58.4(> 18.0%)	0.30
CL	7.5(< 25.0%), 32.9(> 25.0%)	0.30
ASL	125(< 2.5%), 175(> 5%),325(> 10%),316(> 15%)	0.30
IPSL	43(< 2.5%), 61(> 5%),113(> 10%),110(> 15%)	0.30
OPSL	150(< 2.5%),211(> 5%),391(> 10%),381(> 15%)	0.30
IL	40(< 2.5%), 57(> 5%),105(> 10%),102(> 15%)	0.30
SPL	304(< 2.5%),428(> 5%),792(> 10%),771(> 15%)	0.30
STL	326(< 2.5%),458(> 5%),848(> 10%),826(> 15%)	0.30
Implant		
Coflex	110,000	0.30
Pedicle screw and rod	110,000	0.30

ALL: anterior longitudinal ligament; PLL: posterior longitudinal ligament; SSL: supraspinal ligament; ISL: interspinous ligament; LF: ligamentum flavum; TL: transverse ligaments; CL: capsular ligament; ASL: anterior sacroiliac ligament; IPSL: inner posterior sacroiliac ligament; OPSL: outer posterior sacroiliac ligament; IL: interosseous ligament; SPL: sacrospinous ligament; STL: sacrotuberous ligament

Eight nodes of quadratic tetrahedral (C3D8) elements were assumed to mesh the bone and IVD. Tension truss (T2D2) elements were applied to simulate annulus fibers and ligaments. The FE models included approximately 140,000 elements, 160,000 nodes, and 500,000 degrees of freedom. A mesh convergence test was designed to determine the best mesh resolution for the FE model. On the basis of our previously published model [20–22], the mesh density produced well-converged results with element edge lengths around 1–1.5 mm. When the number of solid elements was doubled in the model, mesh convergence results showed a less than 5% difference in ROMs and disc loads.

### Boundary and loading conditions

A moment of 7.5 Nm in flexion and extension, 7.5 Nm in lateral bending, and 5 Nm in axial rotation was used to simulate physical activity. Two femurs were limited in all models in all degrees of freedom. Calculations were performed using the finite element program ABAQUS (SIMULIA Inc., Providence, Rhode Island, USA).

### Data analysis

SPSS software (IBM Corp, Armonk, NY, USA) was used to analyze the data. The ROM, IDP, and maximum stress of annulus fibrosis in the four type models were measured and recorded after the single- or double-level fixation under different loading conditions. The effect of different single- and double-level fusion techniques on the biomechanical behaviors in the four sagittal lumbopelvic models was analyzed by comparing the simulation results.

## Results

### Validation of the models

**Fig. 4:**
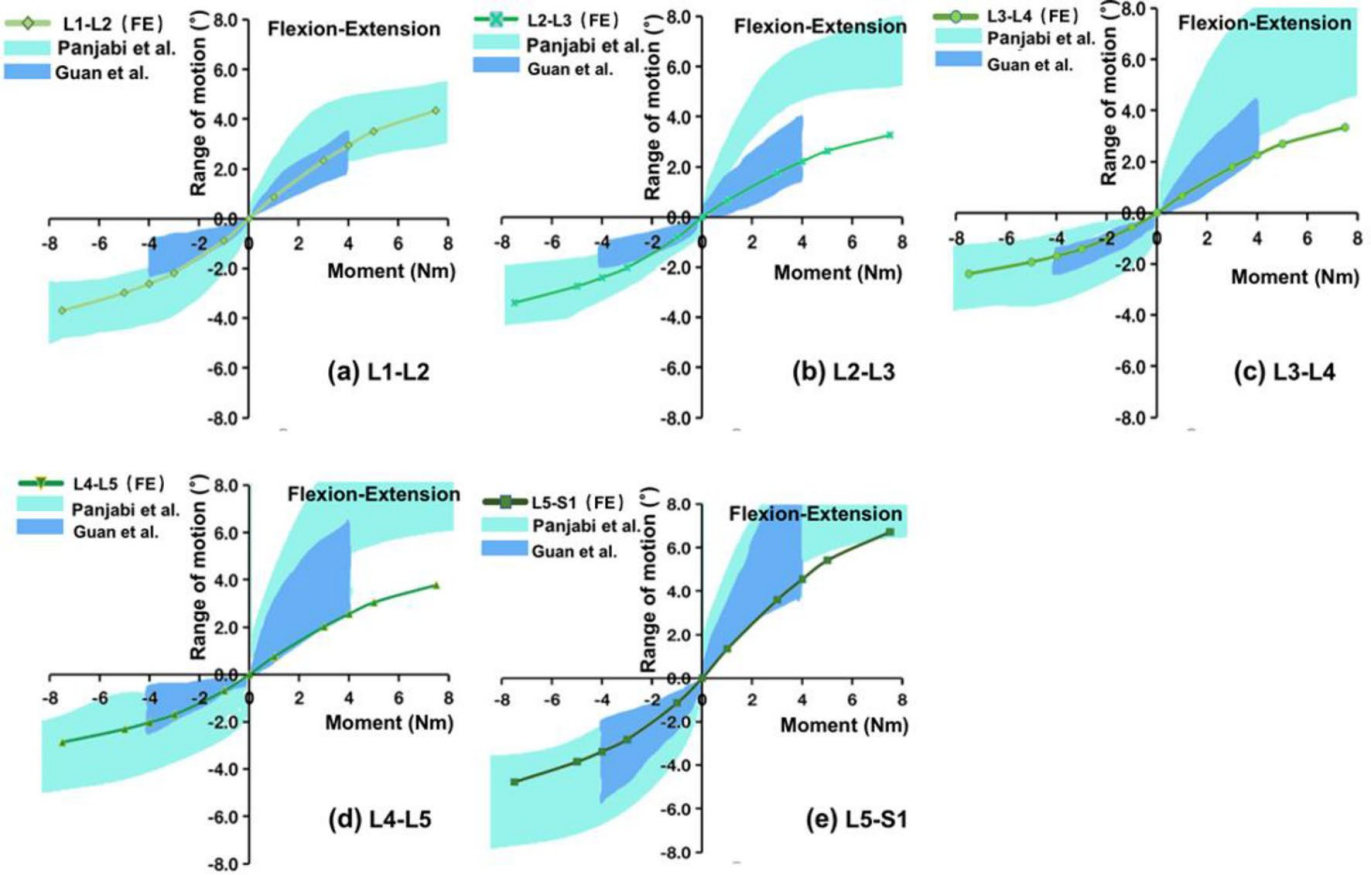
Comparison of the moment-rotation curve between the finite element model and the in vitro experiment in flexion and extension. **a** L1–L2 segment; **b** L2–L3 segment; **c** L3–L4 segment; **d** L4–L5 segment and **e** L5–S1 segment

The calculation results of the intact model before fusions were compared with the in vitro biomechanical experimental data tested by Panjabi [23], Guan [24], and Renner et al. [25], to verify the validation of the model under pure flexion, extension, left–right bending, and left–right rotation loading. The results showed that the moment-rotation curves under the six-moment loading were within the test range reported in the literature (Fig. 4 and Additional file 1: S1, S2). The lumbar–pelvis model was also previously tested in our in vitro experiments by using a robotic device under similar loads as simulated in this study [14]. The moment-rotation behaviors of the finite element model had good agreement with those recorded hysteresis curves in the in vitro experiments, with an overall average error of ~ 6 to ~ 18%.

### Intervertebral rotations in the single-level fixation model

**Fig. 5:**
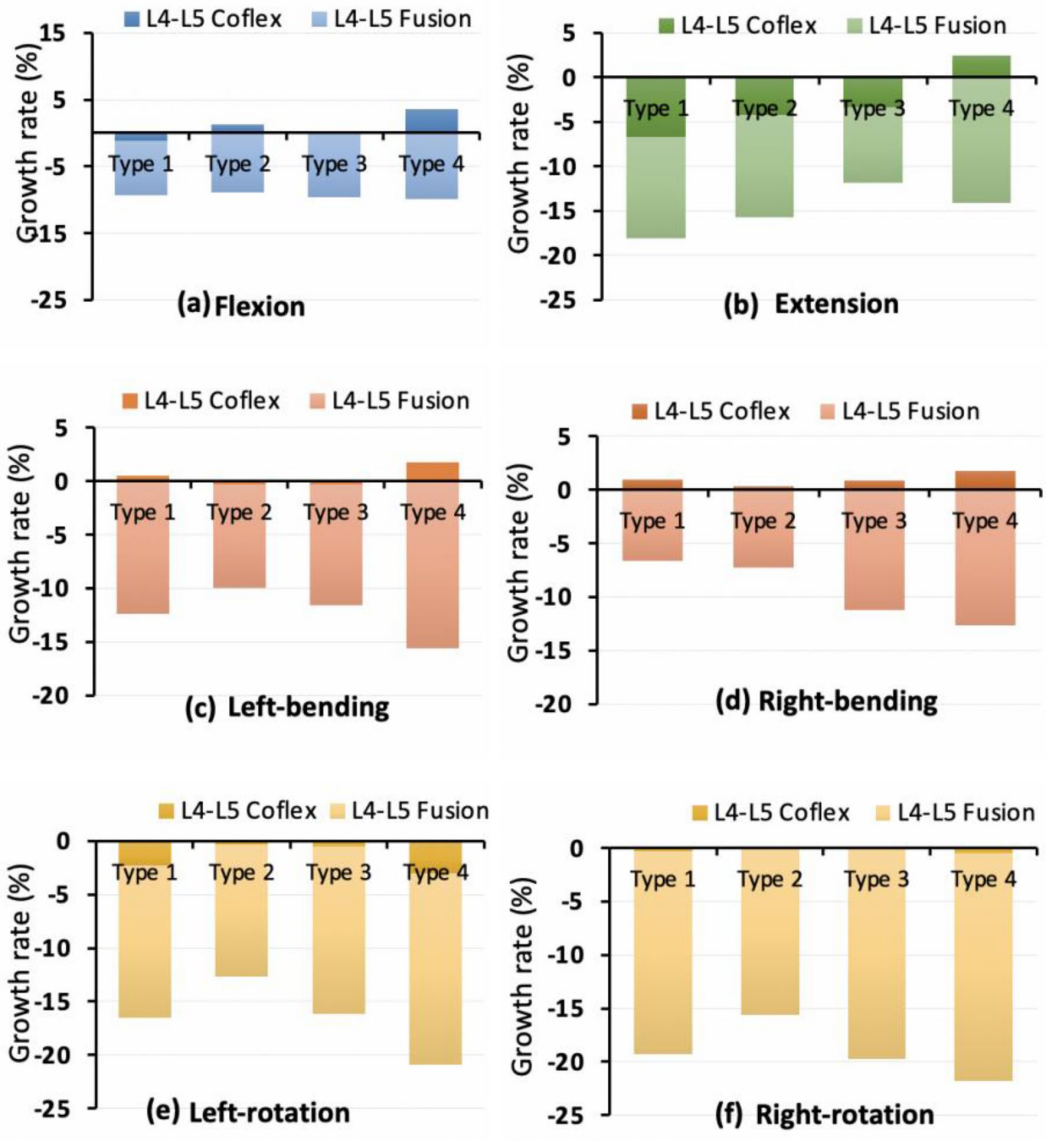
Growth rate of the overall range of motion after the L4–5 single-level fixations

Under the different loading conditions, the L4–l5 Coflex fixation had a minor influence on overall ROM in the four type models, ranging from 0 to 7% (Fig. 5). In extension, the percent decrease in overall ROM in the four type models was higher than that in the other loading conditions, ranging from 2 to 7%. In general, the overall ROM reduction in the L4–L5 Fusion models was around three times that of the L4–L5 Coflex models, ranging from 7 to 21%.

**Fig. 6:**
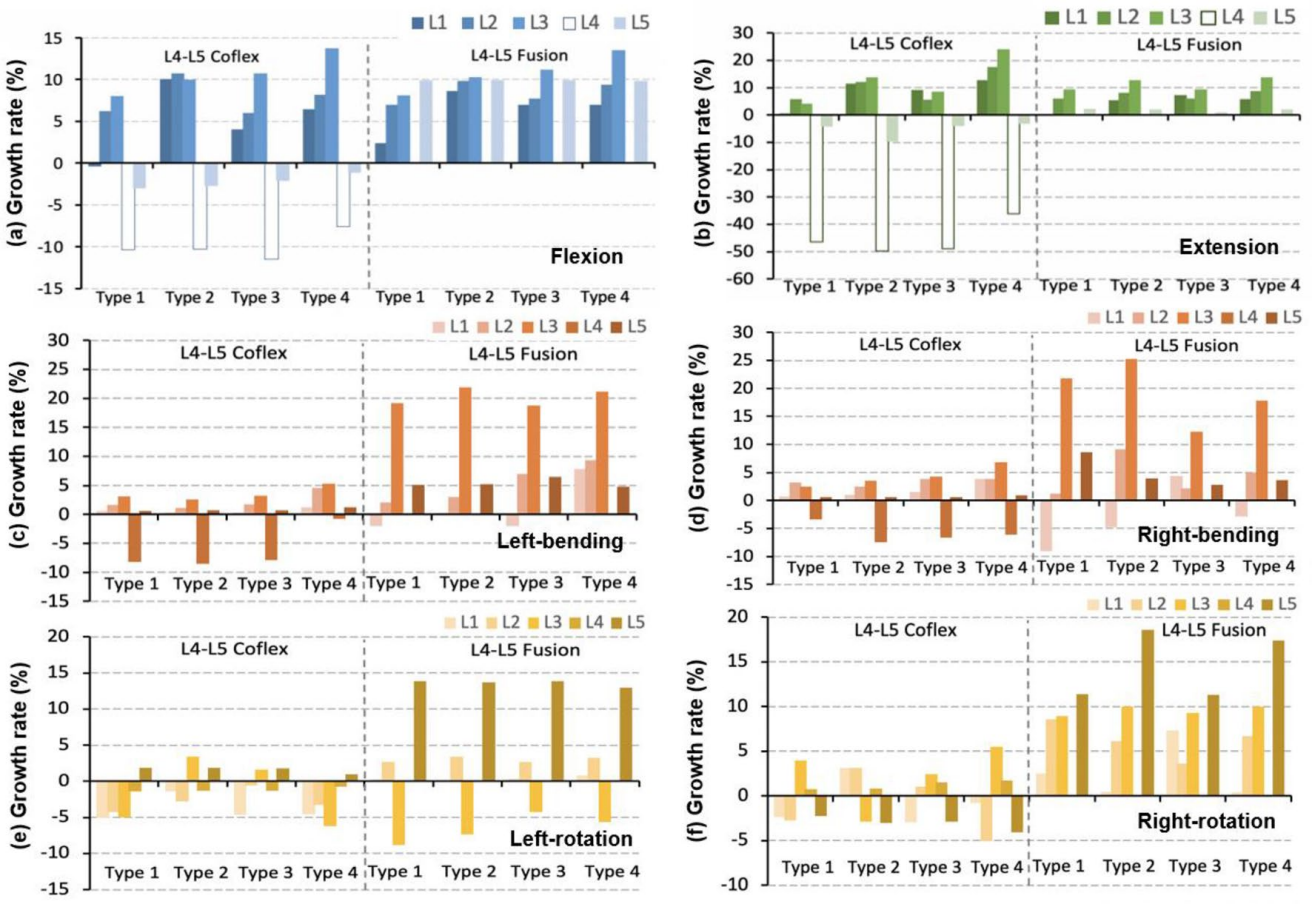
Growth rate of the range of motion at adjacent segments after the L4–5 single-level fixations. L1: L1–L2 segment; L2: L2–L3 segment; L3: L3–L4 segment; L4: L4–L5 segment; L5: L5–S1 segment

Under different loading conditions, the intervertebral rotation for adjacent segments varied in the four type models (Fig. 6). In flexion, the ROM of the L3–4 upper adjacent segment in the L4–5 Fusion models increased by about 1% greater than those in the L4–5 Coflex models (Fig. 6a). In Type 1, Type 2, and Type 3 models, there was no difference in the upper adjacent segments, however, in the Type 4 model, the L4–5 Coflex group had a higher ROM increase in the L1–L4 adjacent segments than that of the L4–5 Fusion group. In extension, there was no difference between the upper adjacent segments of Type 1, Type 2, and Type 3 models in the L4–5 Coflex group, but the ROM at the L1–L4 adjacent segments in Type 4 model was higher than that in the L4–5 Fusion group (Fig. 6b). In lateral bending, the ROM of upper adjacent segments in the L4–5 Coflex group fluctuated between 1% and 5% in the four models, compared to 5% to 20% in the L4–5 Fusion group (Fig. 6c, d). In axial rotation, the adjacent segments changed irregularly in the four models (Fig. 6e, f).

References: As per pubmed findings, citation details for References (4) has been inserted. Kindly check and confirm the inserted details.Correct

### Intradiscal pressures in the single-level fixation model

**Fig. 7:**
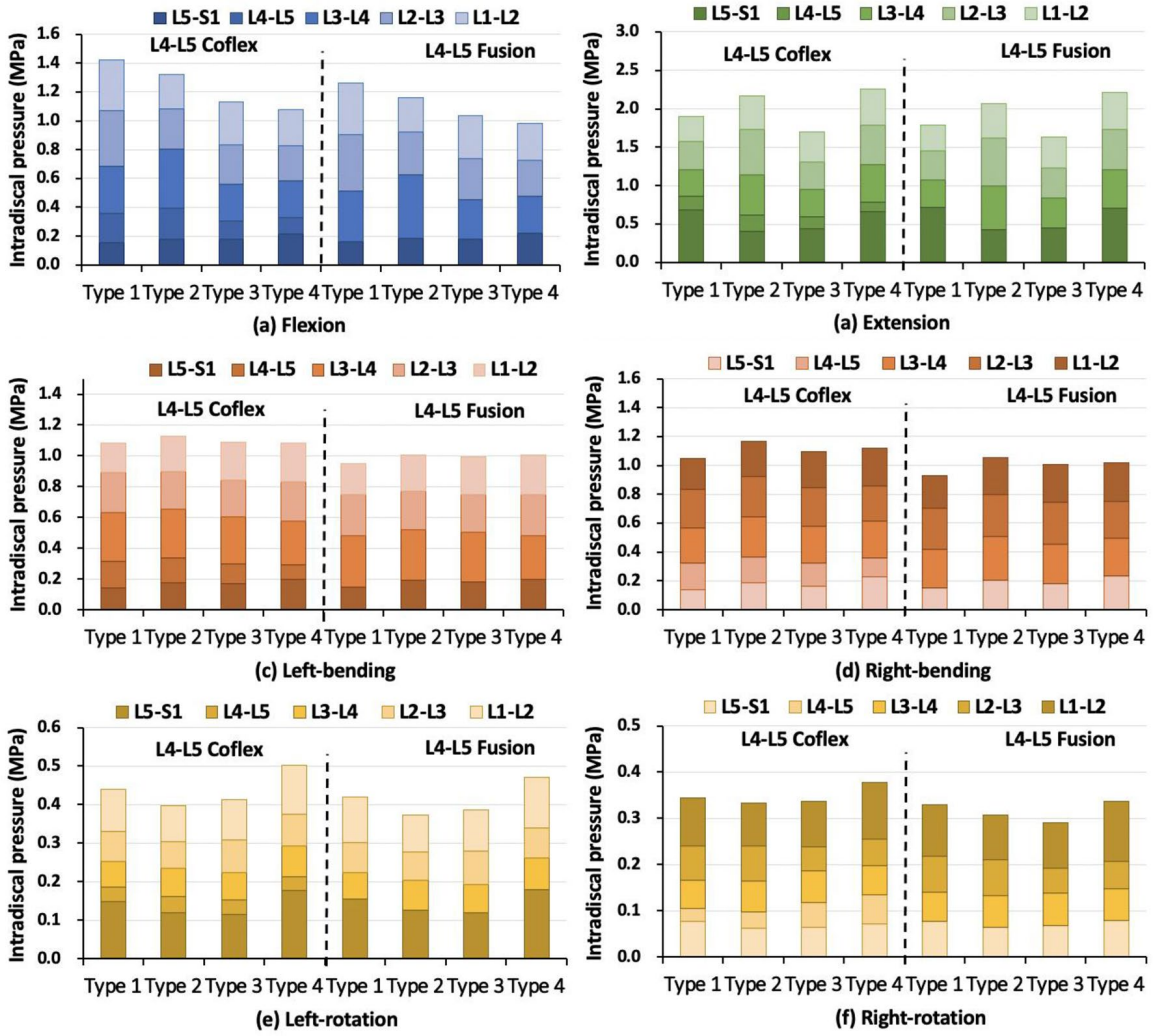
Intervertebral disc pressure at different levels under different loads in four type models after single-level fixations

In flexion, the IDPs at the L3–L4 upper adjacent segments were most affected, and the difference between the L4–5 Coflex and L4–5 Fusion groups was 8% in Type 1 model, 9% in Type 2 model, and 9% in Type 3 model, respectively (Fig. 7a). In extension, the IDP at the L3–L4 level of Type 2 (Coflex: 22% & L4–5 Fusion: 32%) and Type 3 (Coflex: 15% & Fusion: 22%) models were clearly lower in the L4–5 Coflex group than that in the L4–5 Fusion group (Fig. 7b). At the L5–S1 level, the IDPs increased in the L4–5 Coflex (20%-24%) and the L4–5 Fusion groups (26–30%). In lateral bending, the adjacent segments of the four models in the L4–5 Coflex group had a lower IDP increase than that in the L4–5 Fusion group (Fig. 7c, d). In axial rotation, the IDPs of adjacent segments altered irregularly in different models (Fig. 7e, f).

Additional file: As a rule, all supplementary files are to be referred as additional files. Thus, "Supplementary information" was changed to "Additional file 1". Moreover, titles inside the additional files were also amended to correspond with their modified citations. Please check and advise if action taken is appropriate.Correct

### Maximal matrix and fiber stress in the single-level fixation model

**Fig. 8:**
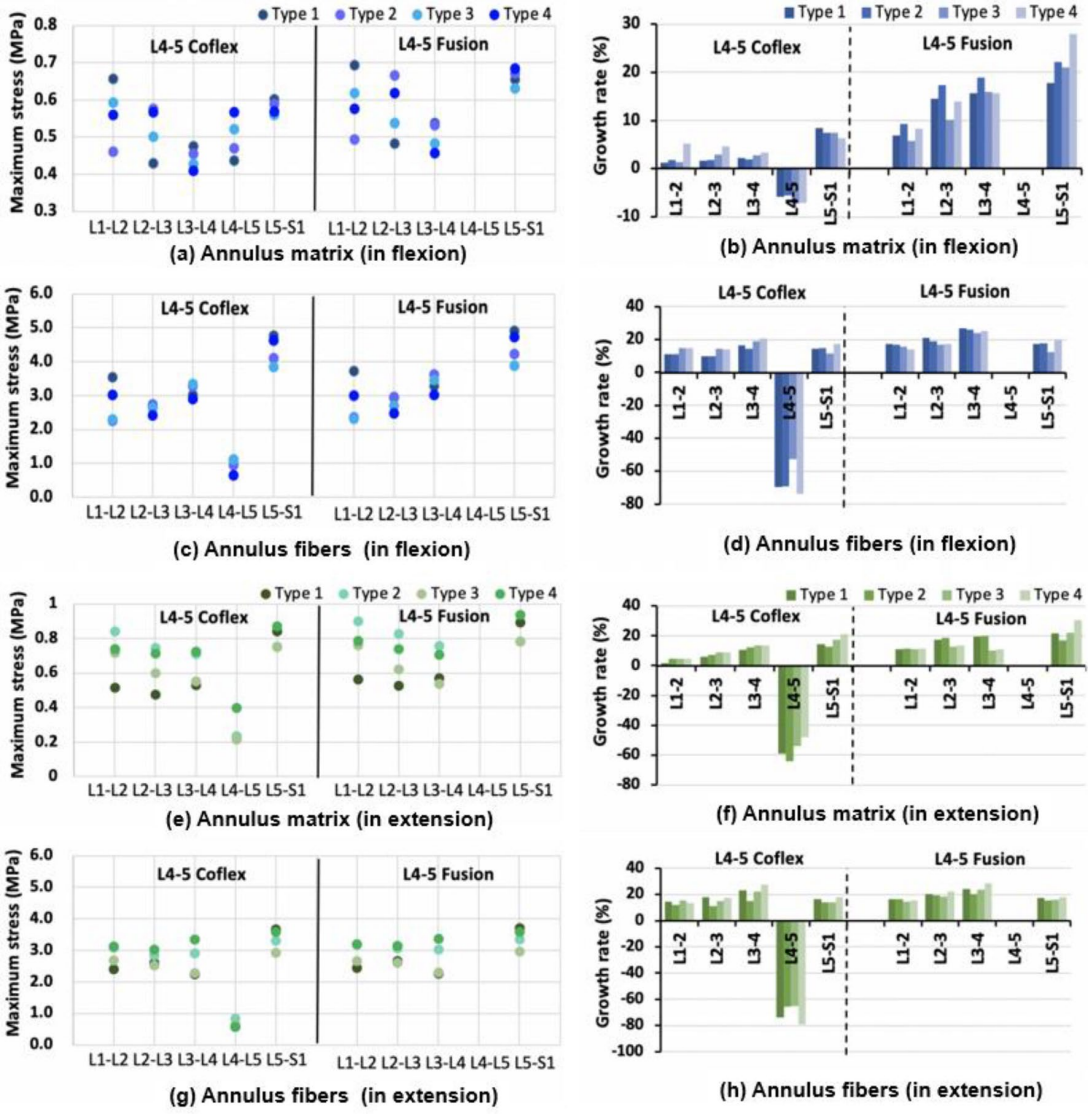
Maximal stress and growth rate of the matrix and fiber at the adjacent segment in four type finite element models after single-level fixation in flexion and extension

The maximal matrix and fiber stress of adjacent segments in the L4–5 Coflex group were generally higher than those in the L4–5 Fusion group (Fig. 8a, Additional file 1: Fig. S3, S4). In flexion, the maximal matrix stress of adjacent segments increased more in the Type 4 model (3% to 5%) than in the other L4–5 Coflex models (1% to 3%) (Fig. 8a). In the Fusion group, the maximal matrix stress of adjacent segments in the Type 2 model increased the most, followed by the Type 1, Type 3, and Type 4 models. In extension, there was less variation in the maximal matrix (2–21%) and fiber (11–28%) stress of adjacent segments between four models in the L4–5 Coflex groups. For the maximum matrix stress, the difference in the adjacent segment after the two fixations in Type 1 and Type 2 models was about 8.5%, more than that of 4.5% in Type 3 and Type 4 models. The difference in maximal fiber stress at adjacent segments was the largest (4–8%) between the two fixations in the Type 2 model compared to that of the other three models (2%). In lateral bending, the maximal matrix and fiber stress at L2–L3 and L3–L4 levels in the Coflex group was larger than that in the Fusion group, while the effect of the two fixations had no difference in the four models (Additional file 1: Fig. S3). In axial rotation, the maximal matrix and fiber stress at adjacent segments showed no regularities (Additional file 1: Fig. S4).

### Intervertebral rotations in the double-level fixation model

**Fig. 9:**
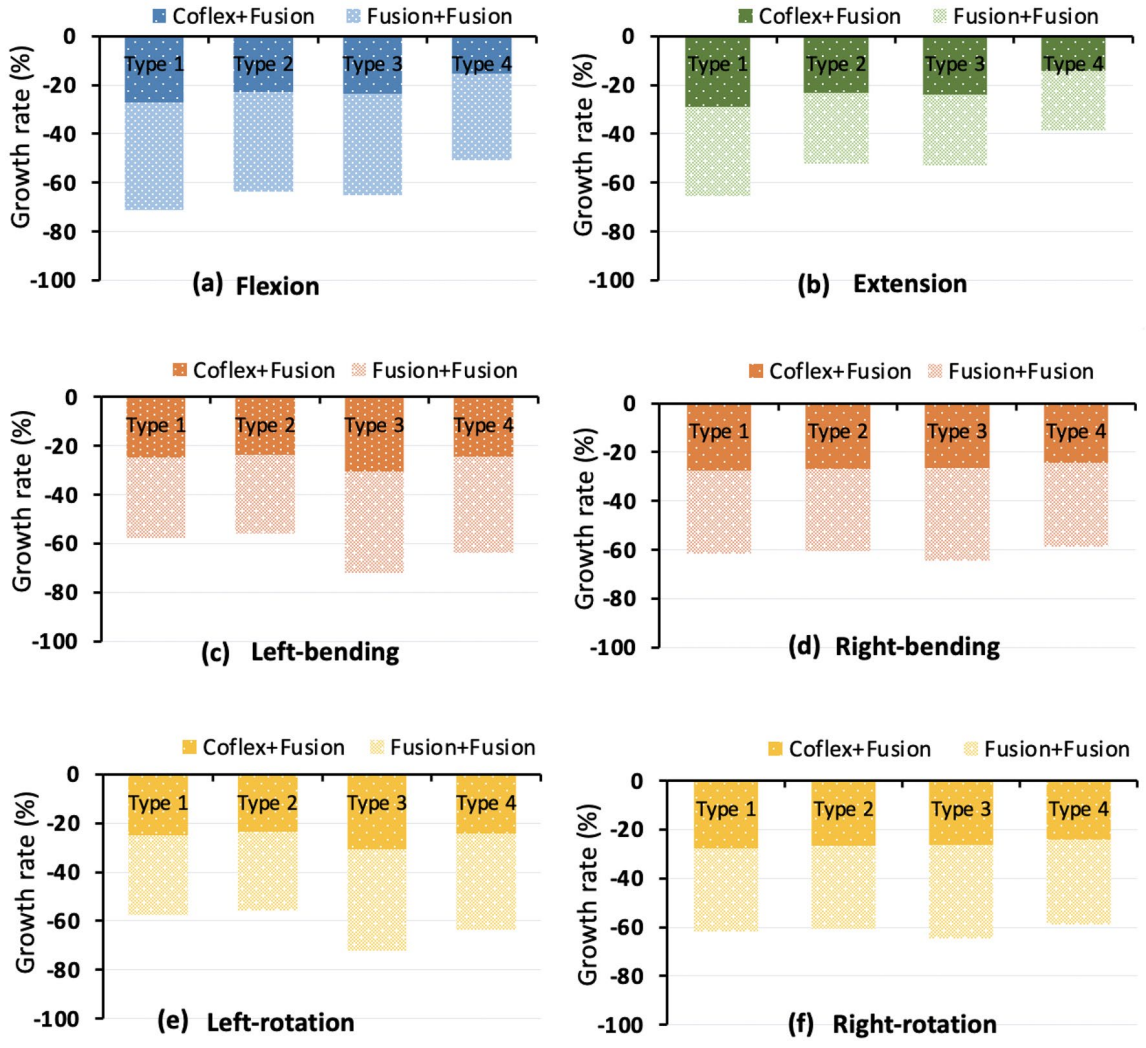
Growth rate of the overall range of motion after the L4–S1 double-level fixations

Under different loading conditions, Coflex + Fusion fixation had a uniform effect on the reduction of the overall ROM of the four models, ranging from 14 to 34% (Fig. 9). In the Fusion + Fusion group, the overall ROM decreased from 25 to 52% about twice that in the Coflex + Fusion group.

**Fig. 10:**
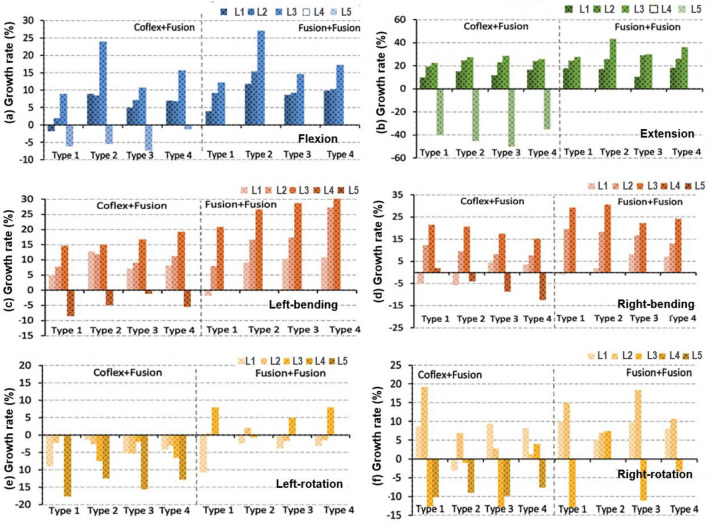
Growth rate of the range of motion at adjacent segments after the L4–5 single-level fixations. L1: L1–L2 segment; L2: L2–L3 segment; L3: L3–L4 segment; L4: L4–L5 segment; L5: L5–S1 segment

In flexion, the effect of the two double fixations showed little difference in the ROM of L3–4 upper adjacent (Fig. 10a). For the L2–L4 adjacent segments, the ROM relative change in Type 4 model in the Coflex + Fusion group was slightly larger than that in the Fusion + Fusion group, while the data in the other three models showed the opposite. The Type 2 model had the largest difference in the relative change of adjacent segments between the Coflex + Fusion and Fusion + Fusion groups, followed by in Type 4, Type 3, and Type 1 models. In extension, the ROM of the adjacent segments in the Coflex + Fusion group increased slightly higher than in the Fusion + Fusion group (Fig. 10b). The difference in the effect of the two double fixations on adjacent segments was similar in the four models. In lateral bending, the increase of adjacent segments in the Coflex + Fusion models was smaller than that in the Fusion + Fusion models (Fig. 10c, d). In axial rotation, the ROM of adjacent segments in the Coflex + Fusion fixed group and Fusion + Fusion fixed group fluctuated randomly in different models (Fig. 10e, f).

### Intradiscal pressures in the single-level fixation model

**Fig. 11:**
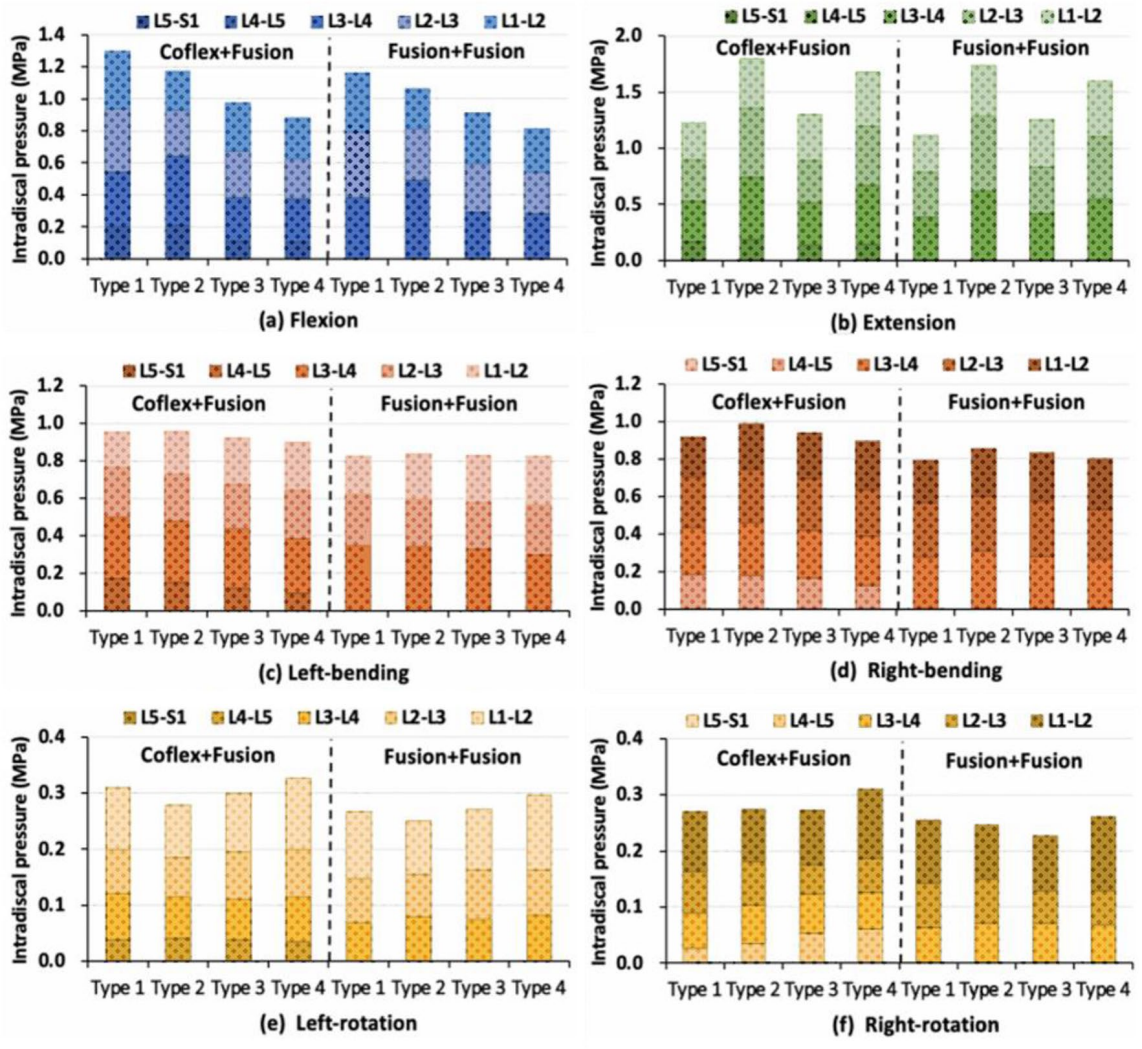
Intervertebral disc pressure at different levels under different loads in four type models after double-level fixations

In flexion, IDPs on adjacent segments in the Coflex + Fusion groups (19–37%) were smaller than those in Fusion + Fusion fixed group (24–43%) (Fig. 11a). The difference between the two double-level fixations was largest in the Type 2 model, followed by Type 1 and Type 3 models, and smallest in the Type 4 model. In extension, the IDP of adjacent segments in Type 2 and Type 3 models was significantly smaller than that in Type 1 and Type 4 models in the Coflex + Fusion group, while there was little difference in the changes of IDPs between the four models in the Fusion + Fusion group (Fig. 11b). The difference in the IDP between the two double-level fixations among the four models was the same as that in flexion. In lateral bending, the adjacent segments in the L4–5 Coflex group had a lower IDP increase (12–26%) than that in the L4–5 Fusion group (15–32%) (Fig. 11c, d). In axial rotation, the IDPs of adjacent segments fluctuated irregularly in different models (Fig. 11e, f).

### Maximal matrix and fiber stress in the double-level fixation model

**Fig. 12:**
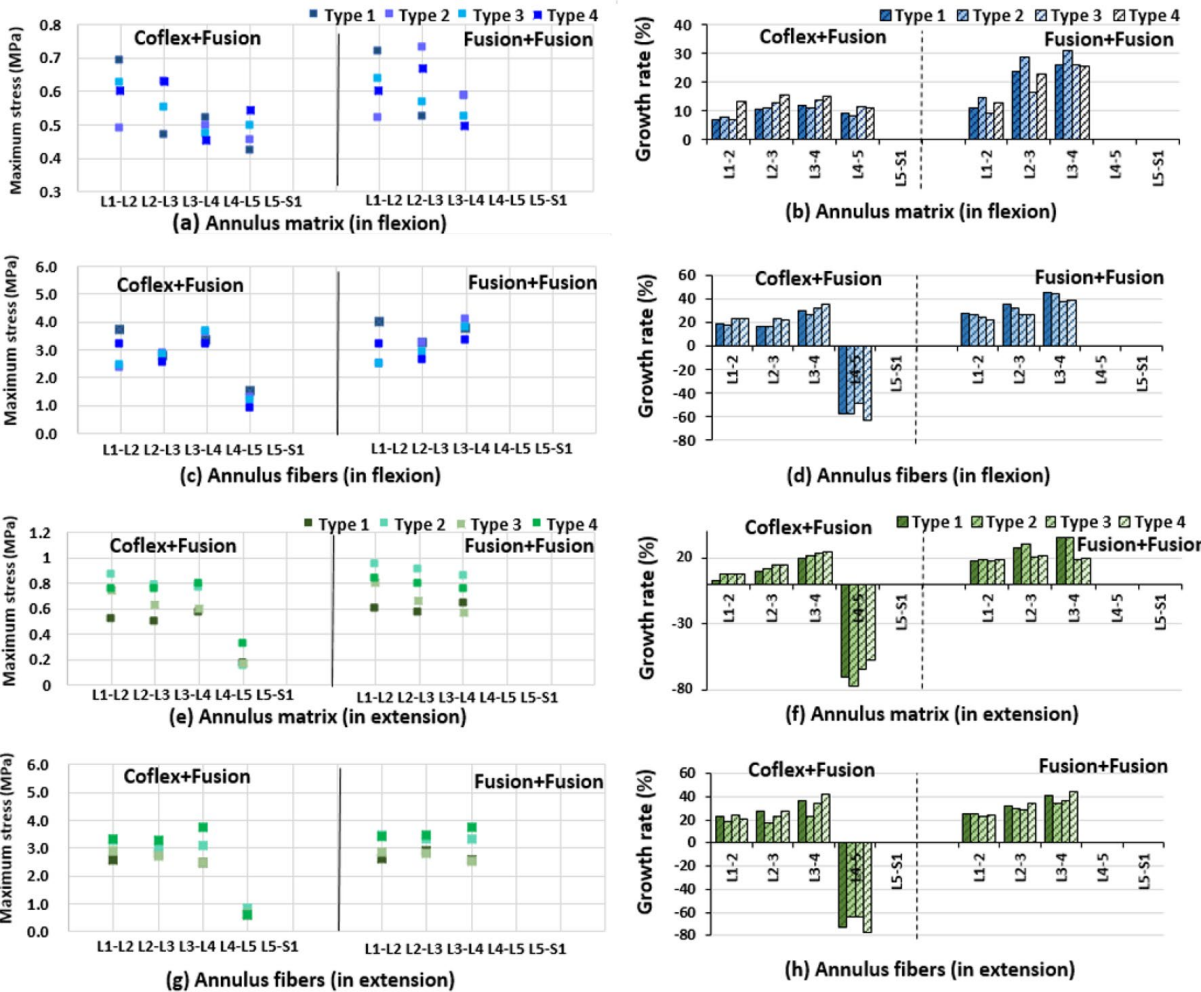
Maximal stress and growth rate of the matrix and fiber at the adjacent segment in four type finite element models after the L4–5 single-level fixations in flexion and extension

The maximal matrix and fiber stress of adjacent segments in the Coflex + Fusion group were generally smaller than those in the Fusion + Fusion group, similar to the results in the single-level fixation (Fig. 12, Additional file 1: Figs. S5, S6). In flexion, the maximal matrix and fiber stress at adjacent segments in Type 3 and Type 4 models increased slightly more than in Type 1 and Type 2 models in the Coflex + Fusion fixed group, whereas the results were the opposite in the Fusion + Fusion group (Fig. 12). The difference between the two double-level fixations in Type 1 and Type 2 models was higher than that of Type 3 and Type 4 models. In extension, the maximal matrix at adjacent segments changed little between the four models in the Coflex + Fusion group, while the maximal fiber stress increased slightly more in Type 1 and Type 4 models than in Type 2 and Type 3 models. In the Fusion + Fusion group, the maximal matrix stress of adjacent segments in Type 1 and Type 4 models was larger than that in Type 2 and Type 3 models, while the maximum fiber stress was larger in Type 1 and Type 4 models (Fig. 12). The difference in maximal matrix stress between the two double-level fixations was 11%–18% in Type 1 and Type 2 models larger than 10% in Type 3 and Type 4 models. In lateral bending, the maximal matrix and fiber stress in the Coflex + Fusion group was lower than in the Fusion + Fusion group (Additional file 1: Fig. S5). In axial rotation, the maximum matrix and fiber stress at adjacent segments fluctuated among the models (Additional file 1: Fig. S5).

## Discussion

Although the dynamic Coflex and traditional posterior pedicle fixation techniques have been validated as effective methods for treating lumbar degenerative diseases, there is still no consensus or guidelines for the surgical procedure. In the past decades, many studies have proved the effectiveness of the sagittal alignment on the biomechanical adaptation and compensation of the spine that is related to implementing and predicting spinal disorders and accurate surgical strategies. The selection of the proper lumbar surgical method cannot ignore the original morphology of the spine. The parametric FE models of Roussouly’s type (1–4) were developed according to the sagittal spinopelvic parameters of the Chinese population that had been published in our previous research. This study evaluated the different kinetic and biomechanical responses of the four classical Roussouly’s types of the spine after different single or double-level spinal fixations under the daily loading conditions, especially in adjacent segments. Both single-level and double-level spinal fixation had the greatest effect on the ROMs, IDPs, and maximum matrix and fiber stress of the lumbar under flexion loading, followed by lateral bending, extension, and axial rotation loading. The upper adjacent segment was most influenced by the implant in all models and took the most compensation from the fixed segment, while this effect decreased with the increase of the distance between adjacent segments. The kinetic and biomechanical responses after single or double-level spinal fixation were within a range in the four Roussouly’s type models.

In the single-level L4–L5 Fusion group, the ROM, IDP, and annulus fibrosus stress of the lumbar were severely affected under different loading conditions, with no significant difference in all four models. In the L4–L5 Coflex group, the changes in lumbar movement and mechanical stress were less than those in the L4–L5 Fusion group, and the differences in adjacent segments between the two surgical methods varied (Additional file 1: Table S1). The Coflex dynamic fixation had the greatest effect on the biomechanical characteristics of the four models under extension loading, a moderate effect under flexion loading, a weak effect under lateral bending loading, and no effect in axial rotation. These findings were similar to the published study by Wilke et al. [26], which found that the Coflex fixation reduced ROM in extension by about 50% compared to the intact lumbar but had no effect on the ROM in flexion, lateral bending, and axial rotation. For the difference in the four sagittal types of the spine, the Coflex dynamic system is more suited for the Type 2 lumbar under flexion and extension loading, which can effectively reduce the relative change of disc pressure and annulus fibrosus stress in adjacent segments compared to the Fusion fixation system. However, for Type 4 lumbar, the Coflex implantation did not have superiority over posterior pedicle fixation, even leading to a larger increase of ROMs at adjacent segments under flexion and extension loading. There was little difference in the relative change of disc pressure and annulus fibrosus stress between the two surgical methods. For Type 1 lumbar, the Coflex dynamic system exhibited certain advantages in preserving the biomechanical features of adjacent segments. In Type 3 lumbar, the difference between the two fixations was not apparent, despite the Coflex maintaining partial motive function and reducing stress increase in adjacent segments.

In terms of double-level fixation, the reduction rate of the overall ROM in the Fusion + Fusion group was about twice as high as that in the Coflex + Fusion group. In most cases, the changes in the movement, intervertebral disc pressure, and annulus fibrosus stress of adjacent segments in the Coflex + Fusion group were less than those in the Fusion + Fusion group. Compared with the single-level Fusion, the changes in postoperative biomechanical characteristics of the lumbar after the double-level Coflex + Fusion and Fusion + Fusion fixation generally increased to varying degrees, while the differences in the influence of the two two-level fixation methods on adjacent segments of the four lumbar models were similar to that of the single-level Fusion. The results showed that the two-level Coflex + Fusion was the friendliest for Type 2 lumbar and had the advantage of reducing the mechanical changes of adjacent levels for Type 1 lumbar (Additional file 1: Table S2). However, for Type 3 and Type 4 lumbar, due to the large curvature of the lordotic curve of the lumbar itself, it may have a strong compensatory advantage on adjacent segments, and then the influence of both the Coflex + Fusion system and the Fusion + Fusion system on them could be reduced. The index segment of the lumbar had higher stability after the Fusion + Fusion fixation, despite the coflex + fusion combined system partially preserving the mechanical characteristics of adjacent segments.

The Coflex dynamic fixation is thought to be a way of gradually transitioning from fixed to mobile segments, theoretically delaying the acceleration of adjacent segment degeneration caused by lumbar fusion fixation [27]. This study showed that the Coflex system effectively recovered the neutral equilibrium of the lumbar and minimized stress concentration in adjacent segments, preventing the occurrence of ASD. However, as compared to the traditional posterior pedicle fixation techniques, the Coflex has a narrower range of indications, highly dependent on the severity of the patient’s condition. Regardless of whether the Coflex is used in single-level fixation or in double-level fixation combined with pedicle fixation, our study suggested that it may be superior for the straight lumbar, such as Type 2, rather than the hypolordotic lumbar, such as Type 4. The excellent compensating ability of the larger lordotic Type 3 and Type 4 lumbar could lead to a wider choice of surgical options. The surgical options for small lordotic Type 1 and Type 2 lumbar are, however, more limited and severe. Furthermore, as the fixed levels are increased, the difference between surgical methods will be reduced.

There are several limitations to this study. First, the model was recreated using primarily data from Asian individuals, with no consideration given to physical differences between Caucasian and African populations. Second, we assumed that our model's structural and material properties were typical of a healthy human spine. For comparison, the spine's real structure and materials (including degenerative discs and degenerative disc Implantation) were not optimized or simulated. Future research should look into the evolution of the four models' biomechanical responses at different phases of degeneration. Third, in the follow-up research, most muscles modeled as pure forces should be included and evaluated. Despite these limitations, computer simulations can provide insights into the different kinetic and biomechanical responses of the four classical Roussouly spine types after different single or double-level spinal fixations, as well as a better understanding of how to make an optimal surgical plan based on the patient's morphology.Additional file: As per journal requirements, every additional file must have a corresponding caption. In this regard, please be informed that the caption of Additional file (1) was taken from the additional e-file itself. Please advise if action taken appropriate and amend if necessary.Correct

## Conclusion

Our findings show that different Roussouly sagittal alignment morphotypes have varied biomechanical characteristics after single or double-level lumbar fixation with dynamic Coflex and rigid fusion devices under simulated physiological loading conditions. For the L4–L5 single-level fixation, Coflex dynamic Implantation for Type 1 and Type 2 lumbar preserved the mechanical properties of adjacent levels, whereas Fusion fixation was suggested to be a good choice for patients with Type 4 lumbar. Similarly, for the L4–S1 double-level fixation, the combined Coflex + Fusion also showed some advantages in decreasing mechanical changes of adjacent levels for Type 1 and Type 2 lumbar. The findings can help us understand the effects of various surgical implantation on the biomechanical response of individuals with varied lumbar morphotypes, which could be used to select safer surgical techniques and further explore degenerative mechanisms of adjacent segments.

### Supplementary Information

**Additional file 1:** **Figure S1.** Comparison of the moment-rotation curve between the finite element model and the in vitro experiment in lateral bending. **a** L1–L2 segment; **b** L2–L3 segment; **c** L3–L4 segment; **d** L4–L5 segment and **e** L5–S1 segment. **Figure S2.** Comparison of the moment-rotation curve between the finite element model and the in vitro experiment in axial rotation. **a** L1–L2 segment; **b** L2–L3 segment; **c** L3–L4 segment; **d** L4–L5 segment and **e** L5–S1 segment. **Figure S3.** Maximal stress and growth rate of the matrix and fiber at adjacent segment in four type finite element models after single-level fixation in lateral bending. **Figure S4.** Maximal stress and growth rate of the matrix and fiber at adjacent segment in four type finite element models after single-level fixation in axial rotation. **Figure S5.** Maximal stress and growth rate of the matrix and fiber at adjacent segment in four type finite element models after double-level fixation in lateral bending. **Figure S6.** Maximal stress and growth rate of the matrix and fiber at adjacent segment in four type finite element models after double-level fixation in axial rotation. **Table S1.** Maximal increment of adjacent segments in four types of finite element models after single-segment fusion (%). **Table S2.** Maximal increment of adjacent segments in four types of finite element models after single-segment fusion (%).

## Data Availability

Not applicable.
