# Supplementary material for: Effects of dynamic and rigid implantation on biomechanical characteristics of different sagittal alignment lumbar after single- or double-level spinal fixations: a finite-element modeling study
Source: Eur J Med Res. 2023 Dec 11;28:583. doi: 10.1186/s40001-023-01475-y (PMC10712158; doi:10.1186/s40001-023-01475-y)
Supplement: Supplementary file 1 — Additional file 1: Figure S1. Comparison of the moment-rotation curve between the finite element model and the in vitro experiment in lateral bending. a L1–L2 segment; b L2–L3 segment; c L3–L4 segment; d L4–L5 segment and e L5–S1 segment. Figure S2. Comparison of the moment-rotation curve between the finite element model and the in vitro experiment in axial rotation. a L1–L2 segment; b L2–L3 segment; c L3–L4 segment; d L4–L5 segment and e L5–S1 segment. Figure S3. Maximal stress and growth rate of the matrix and fiber at adjacent segment in four type finite element models after single-level fixation in lateral bending. Figure S4. Maximal stress and growth rate of the matrix and fiber at adjacent segment in four type finite element models after single-level fixation in axial rotation. Figure S5. Maximal stress and growth rate of the matrix and fiber at adjacent segment in four type finite element models after double-level fixation in lateral bending. Figure S6. Maximal stress and growth rate of the matrix and fiber at adjacent segment in four type finite element models after double-level fixation in axial rotation. Table S1. Maximal increment of adjacent segments in four types of finite element models after single-segment fusion (%). Table S2. Maximal increment of adjacent segments in four types of finite element models after single-segment fusion (%). [file 40001_2023_1475_MOESM1_ESM.docx]

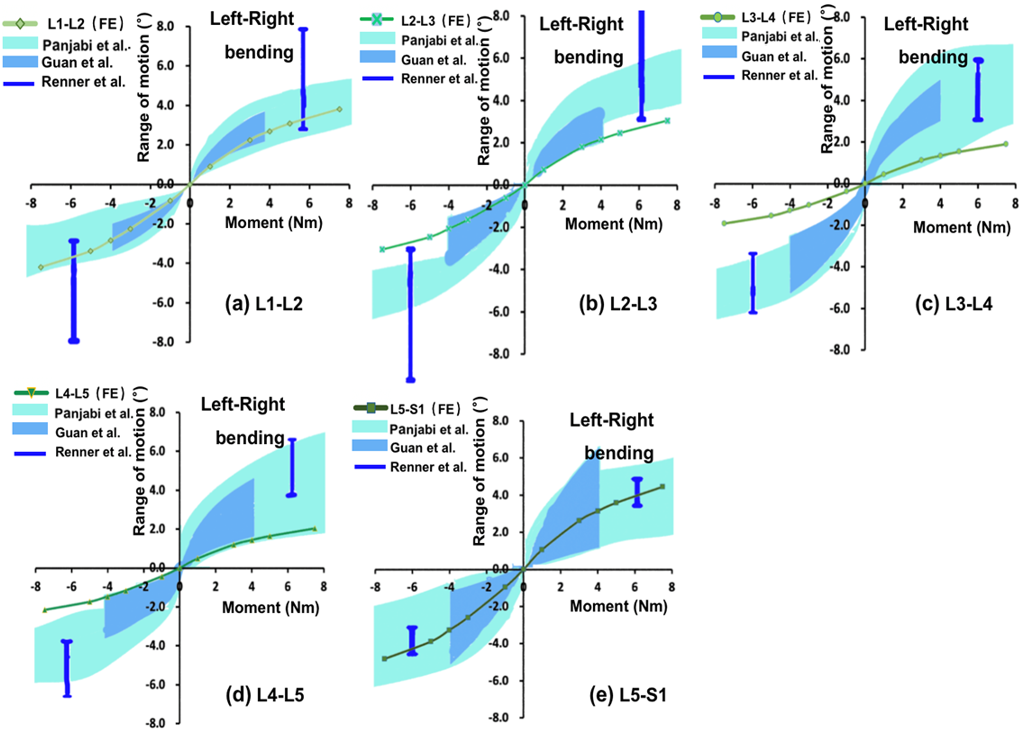


**Figure S1** Comparison of the moment-rotation curve between the finite element model and the in-vitro experiment in lateral bending. (a) L1-L2 segment; (b) L2-L3 segment; (c) L3-L4 segment; (d) L4-L5 segment and (e) L5-S1segment.


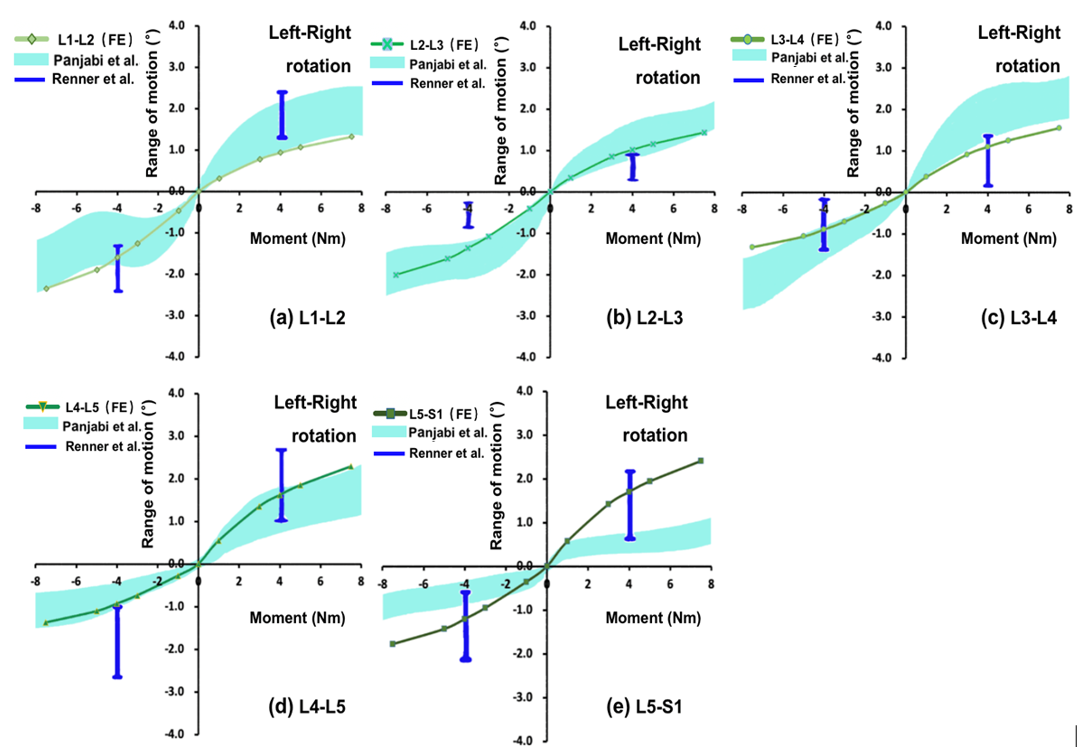


**Figure S2** Comparison of the moment-rotation curve between the finite element model and the in-vitro experiment in axial rotation. (a) L1-L2 segment; (b) L2-L3 segment; (c) L3-L4 segment; (d) L4-L5 segment and (e) L5-S1segment.


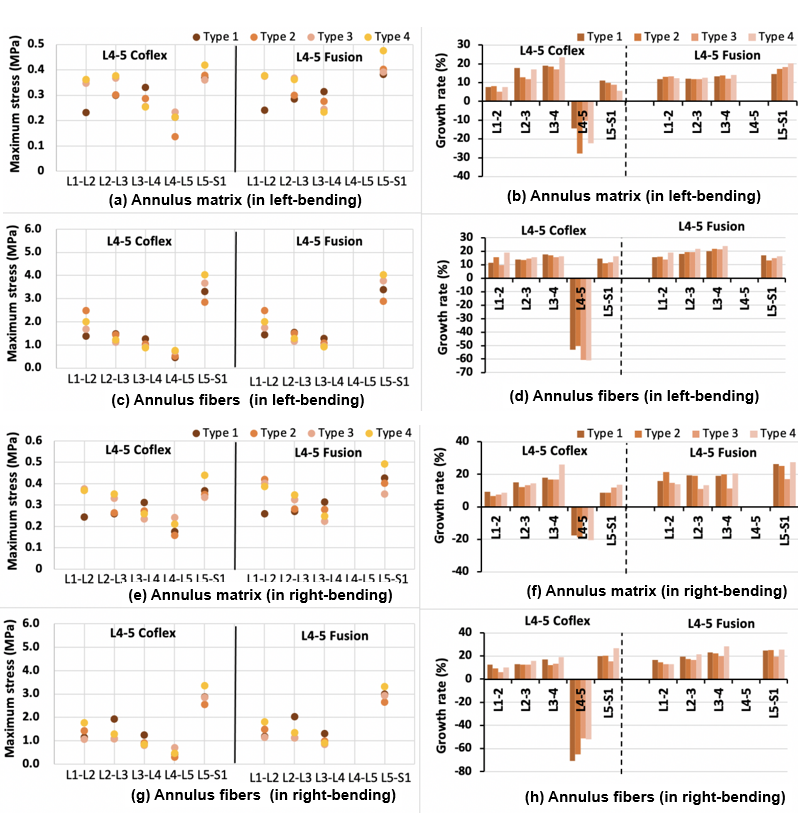


**Figure S3** Maximal stress and growth rate of the matrix and fiber at adjacent segment in four type finite element models after single-level fixation in lateral bending


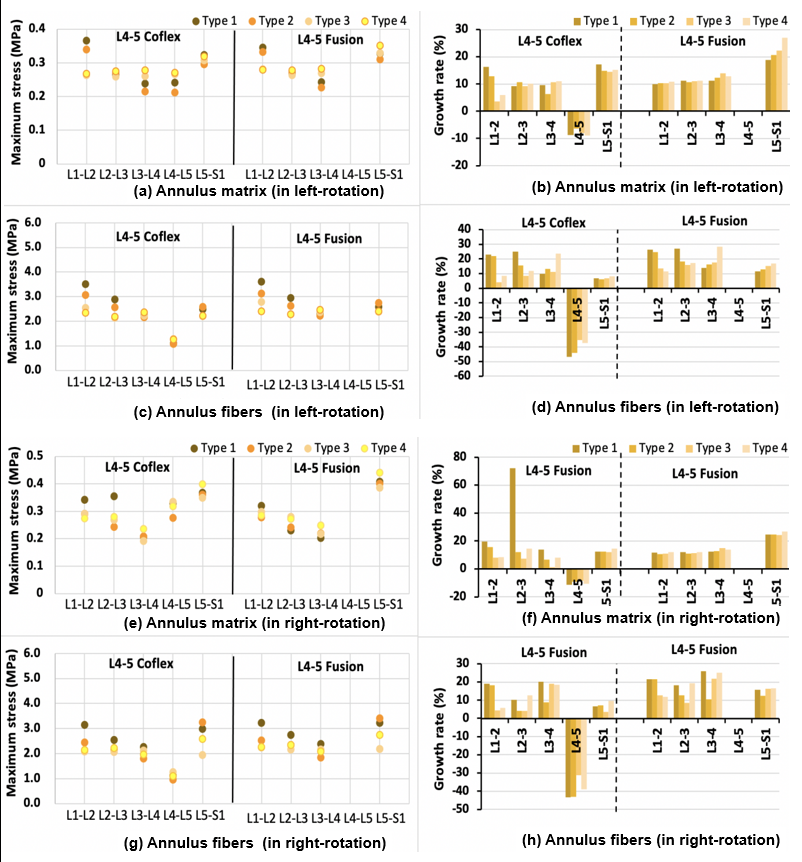


**Figure S4** Maximal stress and growth rate of the matrix and fiber at adjacent segment in four type finite element models after single-level fixation in axial rotation


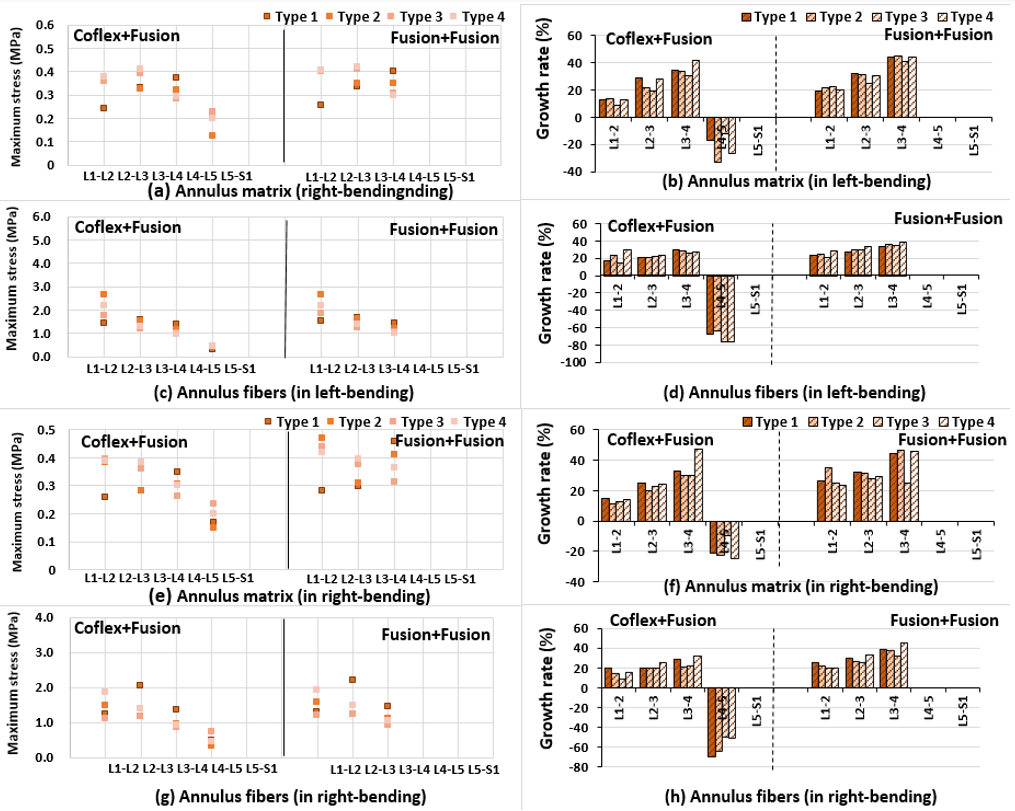


**Figure S5** Maximal stress and growth rate of the matrix and fiber at adjacent segment in four type finite element models after double-level fixation in lateral bending


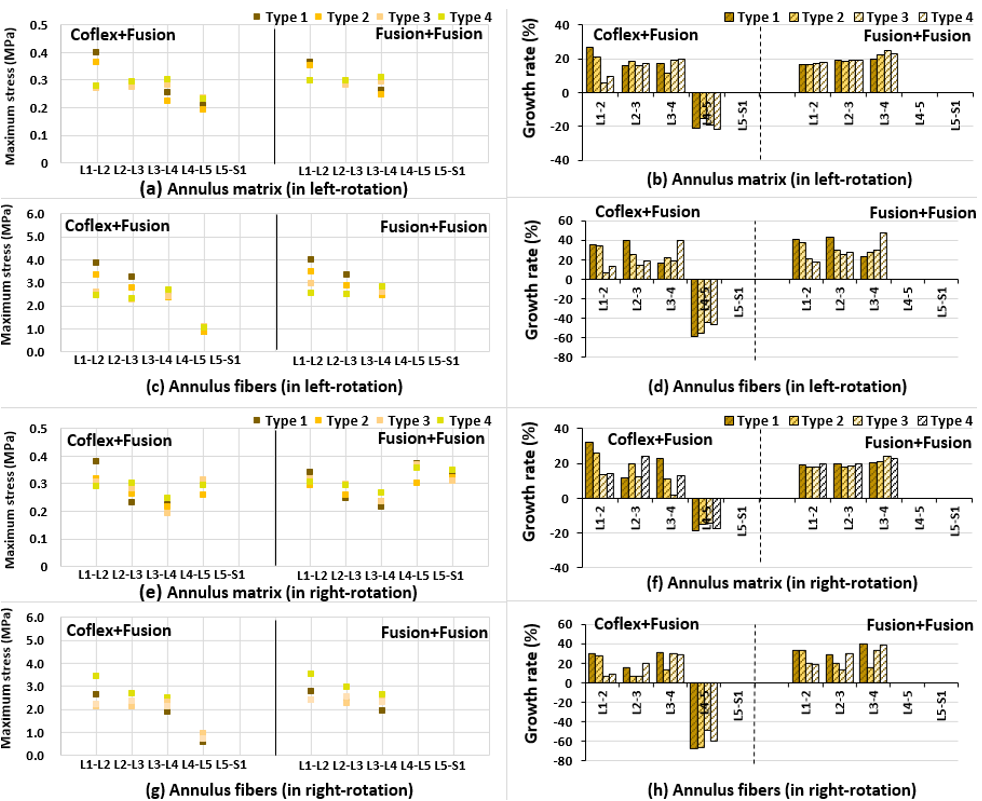


**Figure S6** Maximal stress and growth rate of the matrix and fiber at adjacent segment in four type finite element models after double-level fixation in axial rotation

Table S1 Maximal increment of adjacent segments in four types of finite element models after single-segment fusion（%）

|  |  | **Type 1** | | **Type 2** | | **Type 3** | | **Type 4** | |
| --- | --- | --- | --- | --- | --- | --- | --- | --- | --- |
|  |  | **L4-L5 Coflex** | **L4-L5 Fusion** | **L4-L5 Coflex** | **L4-L5 Fusion** | **L4-L5 Coflex** | **L4-L5 Fusion** | **L4-L5 Coflex** | **L4-L5 Fusion** |
| **Rang of motion** | **flexion** | 8.03% | 8.15% | 9.97% | 10.27% | 10.71% | 11.17% | 13.72% | 13.50% |
|  | **extension** | 14.12% | 9.49% | 13.89% | 12.84% | 8.51% | 9.34% | 24.17% | 13.91% |
| **Intradiscal pressure** | **flexion** | 18.56% | 26.25% | 20.39% | 29.025% | 19.54% | 26.04% | 27.75% | 28.94% |
|  | **extension** | 29.52% | 35.23% | 21.84% | 31.84 % | 14.80% | 21.80 % | 30.23% | 35.23% |
| **Maximal matrix stress** | **flexion** | 2.26% | 15.73% | 1.89% | 18.90% | 2.81% | 15.92% | 3.37% | 15.61% |
|  | **extension** | 10.70% | 19.49% | 12.22% | 19.90% | 13.43% | 10.15% | 13.71% | 10.92% |
| **Maximal fiber stress** | **flexion** | 16.54% | 26.72% | 14.31% | 26.00% | 19.14% | 24.05% | 20.62% | 25.05% |
|  | **extension** | 23.42% | 24.26% | 15.20% | 20.25% | 22.43% | 23.48% | 27.56% | 28.53% |

Table S2 Maximal increment of adjacent segments in four types of finite element models after single-segment fusion（%）

|  |  | **Type 1** | | **Type 2** | | **Type 3** | | **Type 4** | |
| --- | --- | --- | --- | --- | --- | --- | --- | --- | --- |
|  |  | **Coflex+Fusion** | **Fusion+Fusion** | **Coflex+Fusion** | **Fusion+Fusion** | **Coflex+Fusion** | **Fusion+Fusion** | **Coflex+Fusion** | **Fusion+Fusion** |
| **Rang of motion** | **flexion** | 22.38% | 27.75% | 27.49% | 43.56% | 28.72% | 30.15% | 25.92% | 36.17% |
|  | **extension** | 8.92% | 12.23% | 24.00% | 27.1% | 10.71% | 14.64% | 15.68% | 17.25% |
| **Intradiscal pressure** | **flexion** | 24.05% | 37.50% | 26.84% | 43.59% | 26.10% | 35.60% | 37.47% | 42.63% |
|  | **extension** | 33.80% | 46.27% | 29.41% | 44.92%、 | 19.49% | 32.72% | 39.30% | 46.48% |
| **Maximal matrix stress** | **flexion** | 11.90% | 25.95% | 11.26% | 31.18% | 13.91% | 26.27% | 15.09% | 25.75% |
|  | **extension** | 19.27% | 35.09% | 22.00% | 35.82% | 23.78% | 18.27% | 24.26% | 19.65% |
| **Maximal fiber stress** | **flexion** | 30.11% | 44.89% | 26.04% | 43.68% | 32.16% | 37.04% | 34.65% | 38.57% |
|  | **extension** | 36.07% | 40.75% | 23.41% | 34.02% | 34.54% | 36.15% | 42.45% | 43.94% |
